# Identification of *Trueperella pyogenes* Isolated from Bovine Mastitis by Fourier Transform Infrared Spectroscopy

**DOI:** 10.1371/journal.pone.0104654

**Published:** 2014-08-18

**Authors:** Samy Nagib, Jörg Rau, Osama Sammra, Christoph Lämmler, Karen Schlez, Michael Zschöck, Ellen Prenger-Berninghoff, Guenter Klein, Amir Abdulmawjood

**Affiliations:** 1 Institut für Pharmakologie und Toxikologie, Justus-Liebig-Universität Gieβen, Gieβen, Germany; 2 Chemisches und Veterinäruntersuchungsamt Stuttgart (CVUAS), Fellbach, Germany; 3 Landesbetrieb Hessisches Landeslabor, Gieβen, Germany; 4 Institut für Hygiene und Infektionskrankheiten der Tiere, Justus-Liebig-Universität Gieβen, Gieβen, Germany; 5 Institut für Lebensmittelqualität und -sicherheit, Stiftung Tierärztliche Hochschule Hannover, Hannover, Germany; CNR, Italy

## Abstract

The present study was designed to investigate the potential of Fourier transform infrared (FT-IR) spectroscopy to identify *Trueperella* (*T.*) *pyogenes* isolated from bovine clinical mastitis. FT-IR spectroscopy was applied to 57 isolates obtained from 55 cows in a period from 2009 to 2012. Prior to FT-IR spectroscopy these isolates were identified by phenotypic and genotypic properties, also including the determination of seven potential virulence factor encoding genes. The FT-IR analysis revealed a reliable identification of all 57 isolates as *T. pyogenes* and a clear separation of this species from the other species of genus *Trueperella* and from species of genus *Arcanobacterium* and *Actinomyces*. The results showed that all 57 isolates were assigned to the correct species indicating that FT-IR spectroscopy could also be efficiently used for identification of this bacterial pathogen.

## Introduction

*Trueperella* (*T.*) *pyogenes*, according to Yassin et al. [1] reclassified from genus *Arcanobacterium* to genus *Trueperella*, is a worldwide known pathogen of domestic ruminants and pigs causing mastitis and a variety of pyogenic infections [2]–[3]. As summarized by Jost and Billington [4] these bacteria are also able to cause diseases in various other animal species. More recently *T. pyogenes* isolated from reptile and camel origin were characterized in terms of pheno- and genotype [5]–[6]. Infections of human patients with *T. pyogenes* are rare [7]–[8]. However, conventional methods for identification of *T. pyogenes*, which are based on biochemical markers such as sugar fermentation, proteolytic activities, haemolytic and CAMP-like haemolytic reactions [2]–[9] are often time consuming. In addition, atypical variants in phenotype might occur [10]–[11]. Alternative methods based on the analysis of species specific regions of the bacterial genome, which could also be used for identification of *T. pyogenes* [9], can be quite elaborate, expensive and often requires highly skilled staff.

As a high resolving phenotypic technique, FT-IR spectroscopy, analyzing the total composition of components of the bacterial cell, has been established as a method for identification of several bacteria, yeasts and other microorganisms [12]–[13], also including *Actinomycetales* [14]–[15]. Offering a wide range of application this technique was used in the present study to identify *T. pyogenes* isolated from bovine mastitis on species level.

## Materials and Methods

A total 57 *T. pyogenes* isolates obtained from milk samples were used in this study. The bacteria were collected from 2009 to 2012 during routine diagnostic of 55 cows with clinical mastitis from 50 farms and 49 locations. The *T. pyogenes* from milk samples were identified as described previously [6]–[9] by phenotypical and genotypical tests, also including the determination of seven potential virulence factor encoding genes. The studies involved no human or animal participants. The milk samples were taken during routine diagnostic according to national and international guidelines. In all cases from each location was a permission to collect and investigate these samples (Table 1). All samples were taken as part of the hessian udder service to detect reasons for frequently occurring mastitis cases in these holdings.

**Table 1:** Phenotypical and genotypical properties of *Trueperella pyogenes* of bovine origin and two *T. pyogenes* reference strains.

Phenotypical properties	*T. pyogenes* (n = 57)	*T. pyogenes* DSM 20594**	*T. pyogenes* DSM 20630**
Hemolysis on sheep blood agar	+	+	+
CAMP-like reaction with*:			
*Staphylococcus aureus* β-hemolysin	+	+	+
*Streptococcus agalactiae*	−	−	−
*Rhodococcus equi*	+	+	+
Reverse CAMP reaction	−	−	−
β-Glucuronidase (β-GUR)	+^1^^,^ 2	+^1^^,^ 2	+^1^^,^ 2
α-Galactosidase (α-GAL)	− (49)^1^, (+)(8)^1^	−1	−1
β-Galactosidase (β-GAL)	+(56)^2^, (+)(1)^2^	+^2^	+^2^
α-Glucosidase (α-GLU)	+^1^^,^ 2	+^1^^,^ 2	+^1^^,^ 2
β-Glucosidase (β-GLU)	−1	−1	−1
N-acetyl- β-Glucosaminidase (β-NAG)	+^2^	+^2^	+^2^
α-Mannosidase	− (54)^1^, (+)(3)^1^	−1	−1
Catalase	−	−	−
Serolysis on Loeffler agar	+	+	+
Caseinase	+	+	+
Starch hydrolysis (amylase)	−(53), +(4)	−	+
Cross reaction with streptococcal serogroup G specific antiserum	+	+	+
**Genotypical properties**			
*T. pyogenes* specific part of gene *sod*A	+	+	+
*T. pyogenes* specific part of ISR	+	+	+
**Genes encoding virulence factors**			
Pylosin encoding gene *plo*	+	+	+
Collagen-binding protein encoding gene *cbpA*	+ (1), −(56)	−	+
Neuraminidase H encoding gene *nanH*	+(39), −(18)	+	+
Neuraminidase P encoding gene *nanP*	+(48), −(9)	+	+
Fimbriae endoding gene *fimA*	+	+	−
Fimbriae endoding gene *fimC*	+(53), −(4)	+	+
Fimbriae endoding gene *fimE*	+(52), −(5)	+	+

The reactions are shown as follows:

* = synergistic CAMP-like reaction with indicator strains;

** = results mostly obtained from Hijazin et al., 2011;

+; positive reaction; (+) = weak reaction −; negative reaction;

1 = tablets containing substrates (Rosco Diagnostica A/S, Taastrup, Denmark);

2 = 4-methylumbelliferyl conjugated substrates (Sigma, Steinheim, Germany).

In addition *T. pyogenes* DSM 20594, *T. pyogenes* DSM 20630^T^, *T. pyogenes* CVUAS 0222, *Trueperella abortisuis* DSM 19515^T^, *Trueperella bernardiae* DSM 9152^T^, *Trueperella bialowiezensis* DSM 17162^T^, *Trueperella bonasi* DSM 17163^T^, *Arcanobacterium haemolyticum* DSM 20595^T^, *Arcanobacterium canis* DSM 25104^T^, *Arcanobacterium hippocoleae* DSM 15539^T^, *Arcanobacterium phocae* DSM 10002^T^, *Arcanobacterium phocae* DSM 10003, *Arcanobacterium phocisimile* DSM 26142, *Arcanobacterium pluranimalium* DSM 18483^T^, *Actinomyces bovis* DSM 43014^T^, *Actinomyces hyovaginalis* CVUAS 4295, *Actinomyces weissii* DSM 24894^T^ and *Actinomyces canis* DSM 15536^T^, were included and used for comparative purposes.

For FT-IR spectroscopy all isolates were cultivated on sheep blood agar for 48 h (+/−0.5 h) at 37°C in 6–10 replicates under microaerobic conditions (GasPak EZ Campy Container System; Becton, Dickinson and Company, Heidelberg, Germany). Harvesting bacterial biomass and preparation of bacterial films on zinc selenide (ZnSe) plates were performed as described previously [16], using an aliquot of 25 µl in a sample zone of a 96 well format ZnSe-plate. Every isolate for the database was measured at least six times using a TENSOR 27 FT-IR spectrometer supplemented with a HTS-XT module (Bruker Optik GmbH, Ettlingen, Germany) in transmission mode from 500 to 4000 cm^−1^ with the coupled software (OPUS 6.5).

The data set for the isolates used for the construction of the differentiation method was divided into two equal parts, as described by Kuhm et al. [16]. The first one, called creation set, was used to create the method. The second one was used to verify the created method to gain the recovery rates (internal recovery set). An internal validation was performed with this internal recovery set.

The 57 well described *T. pyogenes* isolates from bovine mastitis were used for external validation. Results were given as probability for repeated determination, based on the results of the respective internal and external recovery sets (Table 2).

**Table 2:** Validation of genus and species classification by FT-IR, given as probability for correct identification of strains (repeated determinations).

Organism	Isolates	Spectra	n_ref_	n*_val_*	Identification (%)^a^	
					Correct	Incorrect
*Actinomyces* (genus level)	4	36	13	23	95.8	0
*Arcanobacterium* (genus level)	7	87	35	52	92.6	0
*Trueperella* (genus level)	7	89	31	58	95.8	0
*T. abortisuis*	1	15	5	10	96.0	0
*T. bernardiae*	1	13	4	9	100	0
*T. bialowiezensis*	1	19	5	14	91.8	0
*T. bonsai*	1	12	3	9	88.9	0
*T. pyogenes*	3	30	14	16	97.9	0
*T. pyogenes* from milk-samples	57	342	0^b^	342	98.8^b^	0

*n*_ref_, number of spectra used for reference; *n*_val_, number of spectra used for validation. For internal validation, all spectra which had not been included in the reference data set were used.

a The probability of obtaining uncertain results during repeated determinations is given by the residual to 100%.

b For identification of *T. pyogenes*, an external validation was applied using all isolates not included in the reference.

The infrared spectra of the creation set were used in development of the differentiation methods with NeuroDeveloper software (Synthon GmbH, Heidelberg, Germany), which is based on an artificial neural network strategy (ANN) [17]. The second derivatives of the vector-normalized, five-point smoothed spectra of the creation set in the wave number ranges from 2800–3000 cm^−1^ and 500 to 1800 cm^−1^ were used for data analysis in covar mode with a significance of 95%. Four-fifth randomly assorted spectra of the creation set were used as the “training set” of the developer module. With the remaining one-fifth of the spectra, put in the “validation set”, the internal method optimization of wavelength combinations was done [18]. In this way a hierarchical classification scheme is build, consisting of a top level dividing the three genera (*Actinomyces*, *Arcanobacterium* and *Trueperella*), and a subsequent classification level, differentiating the five *Trueperella*-species.

**Figure 1:**
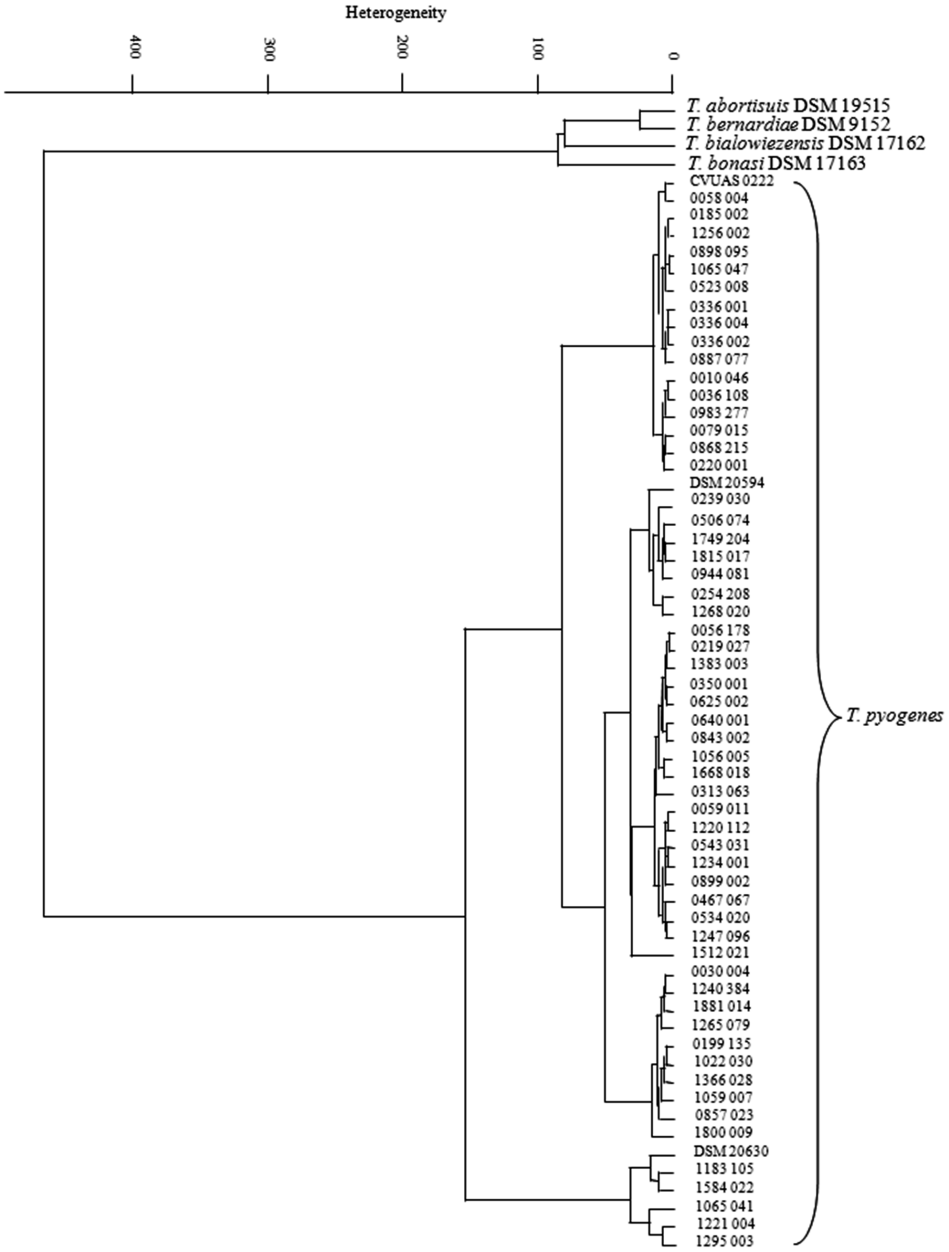
Dendrogram of infrared spectra of 57 *T. pyogenes* isolates from milk samples in comparison with reference strains from the same genus. Cluster analysis was performed by using the second derivatives of the spectra in the spectral ranges of 500 to 1200 cm^−1^ and 2800 to 3000 cm^−1^. Ward's algorithm was applied. The arrows show three independent isolates from the same cow.

Single infrared spectra of all *Trueperella* isolates were compared by cluster analysis as described [15]. For this collation, the second derivatives of vector normalized spectra in the wave number ranges of 500–1200 cm^−1^ and 2800–3000 cm^−1^ were used for calculation with Ward's algorithm (OPUS 4.2) [19]. The dendrogram obtained depicts the arrangement of isolates according to their spectral differences (Fig. 1).

## Results and Discussion

All *T. pyogenes* investigated in the present study were identified, comparable to previously described *T. pyogenes* [5]–[6]–[9], by determination of haemolytic and CAMP-like haemolytic reactions, by using various biochemical tests and genotypically using *T. pyogenes* specific parts of 16S–23S rDNA intergenic spacer region (ISR) [5] and *T. pyogenes* specific parts of superoxide dismutase A encoding gene *sodA* as molecular targets [9]. In addition the amplification of the known and putative virulence factor encoding genes, which had also been previously used to characterize various isolates of this bacterial species [6]–[9], yielded the presence of gene *plo* encoding pyolysin in 57 isolates, gene *cbpA* encoding a collagen-binding protein in one isolate, gene *nanH* encoding neuraminidase H in 39 isolates, gene *nanP* encoding neuraminidase P in 48 isolates and the fimbriae encoding genes *fimA*, *fimC* and *fimE* in 57, 53 and 52 isolates of the investigated isolates, respectively The phenotypical and genotypical properties are summarized in Table 1.

FT-IR spectroscopy, a promising technique for rapid and reliable identification of bacterial microorganisms, had already been used as tool for classification of *Listeria* and *Yersinia* species [16]–, coryneform bacteria [14] and for a large number of other clinically relevant pathogens [15]–[22]–[23]. This spectroscopic technique had also been approved to investigate the most common mastitis-inducing bacteria from genera *Staphylococcus* [24] and *Streptococcus* [25]–[26] and to determine the predominant bacterial flora in raw milk [27]. According to the preliminary results of Prunner et al. [28] FT-IR spectroscopy could also be used to detect *T. pyogenes* in the uterus of cows of Austrian dairy farms.

In order to expand the application of FT-IR spectroscopy for mastitis diagnostics on *T. pyogenes*, a hierarchically structured method based on reference isolates from genus *Trueperella* comprising all type-strains, was created. Additionally, to include taxonomically close relatives cultivated on the same conditions, reference isolates from genera *Arcanobacterium* and *Actinomyces* were integrated in the first level of the hierarchical method. In this first step the isolates were divided into three classes, representing the genus, which were used as a preliminary filter. *Trueperella* isolates were differentiated in a second step down to the species level, distinguishing all recently described members of this genus. For *T. pyogenes* the creation of the FT-IR method succeeded by using three selected isolates (*T. pyogenes* DSM 20594, *T. pyogenes* DSM 20630^T^ and *T. pyogenes* CVUAS 0222). Therewith an adequate segregation of this species from the other species of genus *Trueperella* and the used taxonomically close members of *Arcanobacterium* and *Actinomyces* was achieved (Table 2).

Subsequently FT-IR spectroscopy also allowed the correct classification of the external 57 isolates obtained from bovine clinical mastitis as *T. pyogenes*, with a probability of 98.8% and no error (Table 2). All other bacterial species comprised mostly single isolates. Therefore, a synoptic appraisal for internal validation on genus level was performed. For more than 92% *Arcanobacterium*, *Actinomyces*, and *Trueperella* isolates were assigned to the respective genus correctly.

As mentioned above, only three isolates were required to create the *T. pyogenes*-module. This mirrors the limited intra-species variation of infrared spectra of this species, which can also be shown in the cluster analysis of the *Trueperella* isolates used in this study (Fig. 1). In the dendrogram (Fig. 1) it is shown that the distance of the infrared-spectra of the *T. pyogenes* isolate-variations are far away from all the other known *Trueperella* species, to separate the species *T. pyogenes* unequivocally in this environment (Tab. 2). Noticeable in this context is the clear division of the genus in two branches. One branch comprises the type strains of the four species *T. abortisuis*, *T. bernardiae*, *T. bialowiezensis*, and *T. bonasi*. The second, very close branch was formed by all *T. pyogenes* reference strains, and the 57 isolates from the mastitis cases, independent from the observed variations in phenotype and genotype (Table 1). This shows the slight resolution for single isolates in IR analysis in this isolate set. Up to an exception, the *T. pyogenes* isolates of the present study were obtained from different animals in a wide diversity of farms and locations. Merely three isolates were isolated from one cow. Conspicuously, these three isolates clump together in a special sub-branch (Fig. 1, arrows). Because of the small difference in heterogeneity to the next neighbour isolates, the suitability for a meaningful isolate differentiation cannot be estimated from this limited data-set. In another context, and for other Gram-positive bacteria, like *Bacillus cereus* or *Staphylococcus aureus*, contamination route analysis with FT-IR succeeded by use of FT-IR [29]–[30].

Based on the database containing well defined isolates of the various species of genus *Trueperella, Arcanobacterium* and *Actinomyces* FT-IR spectroscopy, comparable to previously described Matrix-assisted laser desorption-ionization-time of flight mass spectrometry (MALDI-TOF MS) [31], appears to be a promising tool for rapid and reliable identification of *T. pyogenes* in routine diagnosis. In several laboratories FT-IR spectroscopy has become the first choice method for differentiation of various bacterial species. However, FT-IR spectroscopy will not replace but complement the classical phenotypical and genotypical diagnostic systems useful for characterization of *T. pyogenes*.
